# Impact of feedback generation and presentation on self-monitoring behaviors, dietary intake, physical activity, and weight: a systematic review and meta-analysis

**DOI:** 10.1186/s12966-023-01555-6

**Published:** 2024-01-04

**Authors:** Rebecca A. Krukowski, Andrea H. Denton, Laura M. König

**Affiliations:** 1 Department of Public Health Sciences, University of Virginia, PO Box 800765, Charlottesville, VA 22908-0765 USA; 2 University of Virginia, Claude Moore Health Sciences Library, Charlottesville, VA USA; 3 Faculty of Life Sciences: Food, Nutrition and Health, University of Bayreuth, Kulmbach, Germany; 4 Faculty of Psychology, University of Vienna, Vienna, Austria

**Keywords:** Self-monitoring, Diet, Physical activity, Weight, Feedback

## Abstract

Self-monitoring of dietary intake, physical activity, and weight is a key strategy in behavioral interventions, and some interventions provide self-monitoring feedback to facilitate goal setting and promote engagement. This systematic review aimed to evaluate whether feedback increases intervention effectiveness, and which forms of feedback presentation (e.g., personalized vs. not personalized) and generation (i.e., human vs. algorithm-generated) are most effective. To achieve this aim, 5 electronic databases (PubMed/MEDLINE, Web of Science, CINAHL, PsycINFO, and Google Scholar) were searched in April 2022 and yielded 694 unique records, out of which 24 articles reporting on 19 studies were included (with a total of 3261 participants). Two reviewers independently screened titles and abstracts and then full texts and categorized articles as eligible or excluded according to the pre-registered criteria (i.e., availability of full text, peer reviewed manuscript in English; adult participants in a randomized controlled trial that included both self-monitoring and feedback; comparisons of different forms of feedback or comparisons of feedback vs. no feedback; primary outcomes of diet, physical activity, self-monitoring behavior, and/or weight). All included studies were assessed for methodological quality independently by two reviewers using the revised Cochrane risk-of-bias tool for randomized studies (version 2). Ten studies compared feedback to no feedback, 5 compared human- vs. algorithm-generated feedback, and the remaining 4 studies compared formats of feedback presentation (e.g., frequency, richness). A random effects meta-analysis indicated that physical activity interventions with feedback provision were more effective than physical activity interventions without feedback (d=0.29, 95% CI [0.16;0.43]). No meta-analysis could be conducted for other comparisons due to heterogeneity of study designs and outcomes. There were mixed results regarding which form of feedback generation and presentation is superior. Limitations of the evidence included in this review were: lack of details about feedback provided, the brevity of most interventions, the exclusion of studies that did not isolate feedback when testing intervention packages, and the high risk of bias in many studies. This systematic review underlines the importance of including feedback in behavioral interventions; however, more research is needed to identify most effective forms of feedback generation and presentation to maximize intervention effectiveness.

**Trial registration (PROSPERO)**

CRD42022316206.

**Supplementary Information:**

The online version contains supplementary material available at 10.1186/s12966-023-01555-6.

## Background

Overweight and obesity remains a substantial public health challenge worldwide and particularly in higher income countries [1]. Behavioral weight management programs, which use behavioral skills training to help individuals make changes in their eating and activity habits, remain the first-line strategy for overweight and obesity management [2]. Self-monitoring of dietary intake, physical activity, and weight plays a key role in these programs [3, 4], and greater adherence to self-monitoring has been demonstrated to be the best predictor of weight loss success [5–8]. In particular, early and consistent engagement in self-monitoring is important for weight management success [9, 10]. In order to both support greater adherence to self-monitoring and to help individuals interpret the data collected from self-monitoring to set effective behavioral goals, participants in weight management programs are typically provided feedback based on their self-monitoring [11].

Within Social Cognitive Theory, provision of feedback is postulated to provide positive reinforcement for successful goal attainment, insight into potential barriers and challenges, and support for problem-solving and effective development of future goals [12, 13]. Moreover, Supportive Accountability Theory [14, 15] posits that interventionist support is essential for promoting engagement with health behavior change interventions, which often takes the form of feedback. However, beyond these basic theoretical principles, surprisingly little empirical evidence exists to guide the crafting of feedback messages for weight management as well as improvements in dietary intake and physical activity [16].

Feedback has been recognized as a potentially essential ingredient in the behavioral change technique taxonomy [17], and while self-monitoring has been the focus of many systematic reviews/meta-analyses [5, 7, 18, 19], feedback has received less attention [20, 21]. Sherrington and colleagues [20] focused on the personalization of feedback (i.e., individualization of feedback either by a human or an algorithm) in internet-based weight management studies, and they found that personalized feedback may confer approximately a 2 kg benefit over interventions that did not provide personalized feedback. Schembre et al. [21] concentrated on just-in-time feedback in diet and physical activity-focused interventions and was unable to conduct a meta-analysis due to the variability in targeted behaviors, study duration, and feedback types. Thus, it is essential to identify and evaluate feedback types that may optimize this intervention component, given the widespread use of feedback in behavioral interventions for weight management, dietary change and physical activity change, the personnel costs of human-generated feedback [22], as well as the various forms in which feedback may be presented (e.g., positive reinforcement messages vs. areas for change [23]; numerical displays [24, 25] vs. vibrations [26] vs. text [27–29]).

The primary aim of the current study was to systematically review and, if possible, meta-analyze self-monitoring interventions that use feedback as a behavior change technique (BCT), to determine the impact of feedback on diet and physical activity behaviors, weight, and self-monitoring behaviors (i.e., diet or physical activity, or weight). The secondary aims were to evaluate aspects of feedback (e.g., how different types of feedback are perceived by participants, how feedback impacts retention, what types of feedback are typically provided, how frequently feedback is provided, the length of feedback) to determine whether there are potentially feedback elements that are associated with superior outcomes.

## Methods

The review proposal was submitted to PROSPERO prior to data extraction; it was accepted on April 11, 2022, registration number: CRD42022316206. The search strategy, raw data, and analysis scripts are provided on the Open Science Framework (OSF; https://osf.io/j9duf/).

### Search methods for identification of studies

A medical librarian (AD) searched PubMed/MEDLINE, Web of Science, CINAHL, PsycINFO, and Google Scholar. The search was limited to articles published in the English language and published from 1970 through March 2022. Keywords included “self monitor” OR “self monitoring” OR “self monitored” OR “self directed” OR “self evaluate” OR “self regulate” OR “self regulated” OR “self track” OR “self tracking” OR “self weighing” AND obes* OR overweight OR weigh* OR “body mass” OR bmi OR calor* OR diet OR exercise* OR “physical fitness” OR “physical activity” OR walk* OR step OR steps OR pedometer* AND feedback AND behavior* OR behaviour*. Search strategies were modified for each database, utilizing controlled vocabularies (e.g., Medical Subject Headings) as appropriate. Complete search strategies are provided on the OSF website. In addition, searches of reference lists of identified studies and forward citation tracking using Google Scholar was performed by two authors (RAK and LK) to identify further eligible publications.

### Screening

All potentially-eligible study records generated from the search strategy were imported into Covidence systematic review software (Veritas Health Innovation, Melbourne, Australia; available at www.covidence.org). Duplicates were removed before all titles and abstracts were screened independently by two authors (RAK and LK), categorizing articles as provisionally eligible or excluded according to the pre-registered eligibility criteria (Table 1).

**Table 1:** Inclusion and exclusion criteria

	Inclusion criteria	Exclusion criteria
Type of study	Randomized controlled trial; experimental study; peer-reviewed	Literature that was not peer-reviewed (e.g., theses and dissertations); reviews and meta-analyses; conference abstracts that were not published in a full manuscript
Condition or domain being studied	Diet, self-weighing and physical activity behaviors	Exclusive focus on other behaviors
Participants	Adult population (18 years and over; or a mean age within this range)	Children or adolescents under the age of 18
Intervention	Interventions with at least two conditions engaged in self-monitoring, for which feedback was provided related to behaviors or outcomes of behavior	Interventions not including self-monitoring and feedback based on the self-monitoring data
Comparator	At least two experimental groups comparing different forms of self-monitoring feedback (e.g., written vs graphic feedback, different wordings of written feedback) or feedback provision vs. no feedback control that only differ in feedback provision	Groups differ in more aspects than the form of feedback provided or feedback provision
Outcomes	At least one of the following outcomes: Dietary intake; physical activity; self-monitoring diet or physical activity or weight; or weight	Studies focusing exclusively on other outcomes

**Fig. 1:**
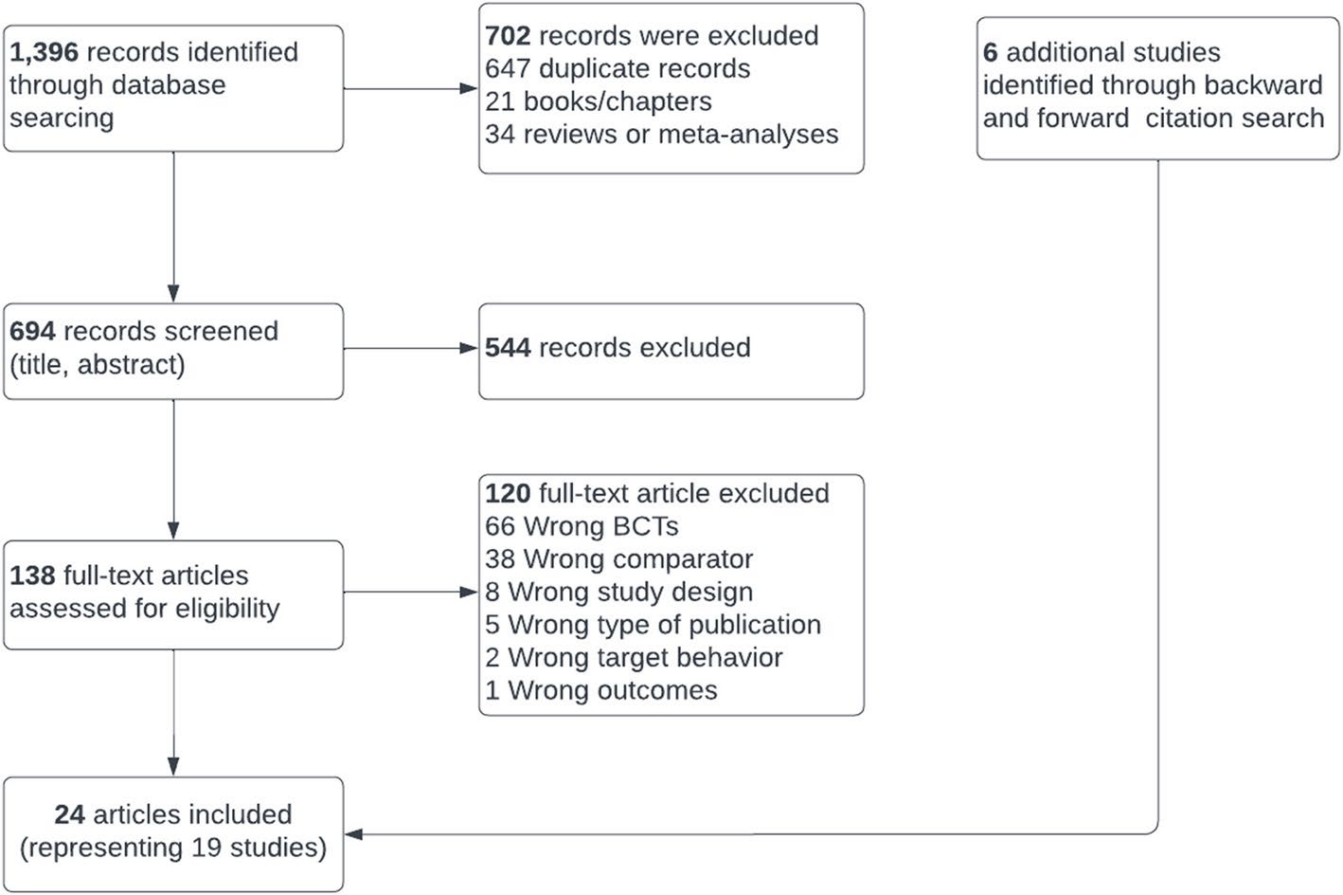
PRISMA Flow Chart

Specifically, articles were evaluated on the following criteria (in order) and categorized as excluded on the first criterion where they did not meet eligibility (if applicable): 1) no full text, 2) not published in English, 3) not an empirical peer-reviewed paper, 4) participants were not adults, 5) not a randomized controlled trial, 6) not an intervention targeting diet, physical activity or self-weighing, 7) BCTs did not include both self-monitoring and feedback (of behavior or outcome of behavior), 8) did not compare different forms of feedback or did not compare 2 or more interventions that only differ in whether feedback is provided, and 9) did not include primary outcomes of diet, physical activity, self-monitoring behavior and/or body weight. Conflicts were resolved by discussion. Afterwards, all full texts were screened independently by the same two authors and coded as eligible or excluded. Again, conflicts were resolved by discussion. The flow of study records is documented in the PRISMA diagram (Fig. 1).

Feedback on behavior and outcomes was defined based on the behavior change technique taxonomy [30]. Specifically, feedback on behavior was defined as “Monitor and provide informative or evaluative feedback on performance of the behavior (e.g., form, frequency, duration, intensity); e.g., inform the person of how many steps they walked each day (as recorded on a pedometer) or how many calories they ate each day (based on a food consumption questionnaire).” Feedback on outcomes was defined as “Monitor and provide feedback on the outcome of performance of the behavior; e.g., inform the person of how much weight they have lost following the implementation of a new exercise regime.”

### Data extraction and synthesis

Two reviewers (RAK and LK) extracted data into a structured coding form. The data extracted included study characteristics (i.e., target behavior(s), country where the intervention took place, inclusion/exclusion criteria, study conditions, sample size, participant characteristics), intervention characteristics (i.e., theoretical foundation, self-monitoring modality, feedback modality, intervention components, self-monitoring duration), feedback characteristics (i.e., frequency, length, type), outcome characteristics, and study results (i.e., effect size for targeted outcomes, overall study conclusions). We also extracted information about feedback perceptions and retention. All relevant study outcomes were included in the extraction and synthesis.

In addition, a meta-analysis was conducted if at least three studies using similar manipulations and reporting on the same outcome provided data on group means and standard deviations or standard errors that could be used to calculate Cohen’s d [31]. We used metafor 3.8–1 [32] in R Studio 2021.09.2/ R version 4.1.2 to compute random effects models to calculate pooled effect sizes and to adjust for potential publication bias using the trim-and-fill method [33]. Heterogeneity was evaluated using I^2^ as recommended by Higgins et al. [34]. To account for multiple comparisons (i.e., when the control group was used for more than one comparison), the N of the control group was split, as recommended by Harrer et al. [35]; this was only the case for one study [36].

### Risk of bias assessment

All studies that were eligible for inclusion were assessed for methodological quality by two reviewers using the revised Cochrane risk-of-bias tool for randomized studies (version 2) [37]. Studies were evaluated related to 6 types of bias: selection bias, performance bias, detection bias, attrition bias, reporting bias, and other sources of bias. Each type of bias is rated as 1) low risk of bias, 2) some concerns, or 3) high risk of bias. For the overall rating, the category indicating the highest risk of bias for an individual component is used. In addition, Egger’s test was conducted to test for publication bias in the meta-analyses [38, 39].

## Results

The literature search yielded 1,396 studies, of which 647 were duplicate citations, 21 were books or chapters and 34 were reviews or meta-analyses, leaving 694 articles to be screened for eligibility. 544 articles were excluded upon title or abstract screening because the study did not meet the inclusion criteria. Thus, 138 full-text articles were assessed for eligibility. After 120 articles that did not meet the inclusion criteria were excluded, there were 18 included publications. An additional 6 studies were identified through forward and backward citation searches. A total of 24 publications reporting on 19 studies were included in the review (see PRISMA diagram, Fig. 1), with a total of 3,261 participants.

### Characteristics of included studies

Of the 19 studies, 6 focused on diet [27, 28, 40–43], 14 focused on physical activity [24–26, 36, 41, 43–52], 3 focused on sedentary behaviors [24, 26, 53], and 9 focused on weight management [24, 27–29, 40–43, 51] (with some studies focusing on more than one of these behaviors) (Table 2). Outcomes for the different behaviors, however, varied widely between studies. Dietary behaviors reported comprised energy intake [27, 41, 47, 54], percent carbohydrates, protein, fat, and saturated fat from total daily energy intake, sodium intake, total fiber, added sugar [54], percent saturated, monounsaturated and polyunsaturated fats from daily energy intake [27], daily vegetable and fruit portions, weekly consumption of sweetened beverages and ultraprocessed foods [43], and achievement of diet goals [40]. Regarding physical activity, studies investigated total minutes of physical activity [50], daily minutes of moderate to vigorous physical activity [43, 51], steps [25, 26, 36, 46], walking lengths [47], physical activity energy expenditure [41, 44], metabolic equivalents [24, 48], accelerometer counts [52], activity data [45], time spend standing [26], sedentary time [26, 53], achievement of physical activity goals [50] (see raw data provided on the OSF).

**Table 2:** Study Characteristics by Comparison Category

Feedback Versus No Feedback											
			Included Sample					Included Conditions			
Study	Target Behavior(s)	Country	Characteristics	Age (M(SD))	% Female/ Women	Ethnicity	N	#	Description	Mode of Self-Monitoring	Duration
Blanson Henkemans et al., 2009 [40]	Weight, diet, physical activity	The Netherlands	Adults with overweight	43.24 (11.55)	82%	N/A	118	2	1) Feedback; 2) No feedback	DieetInzicht website	4 weeks
Burke et al., 2017 [42]	Weight	USA	Adults with overweight/ obesity	44.85 (12.75)	87.18%	84.62% White	26	2	1) Feedback; 2) No feedback	Study-designed app	12 weeks
Burke et al., 2022 [27]	Weight	USA	Adults with overweight/ obesity	45.0 (14.4)	79.50%	84.3% White	502	2	1) Feedback; 2) No feedback	Study-designed app	6 months
Fanning et al., 2017 [51]	Physical activity	USA	Inactive middle-aged adults	41.38 (7.57)	80%	87% White	116	4	1) Goal-setting + Feedback points; 2) Goal setting only; 3) Feedback points only; 4) None	Actigraph accelerometer, study-designed app	12 weeks
Jauho et al., 2015 [24]	Physical activity, weight	Finland	Young men in the military	17.9 (0.7)	0%	N/A	276	2	1) Feedback; 2) No feedback	Polar Active accelerometer	3 months
Lawrie et al., 2018 [45]	Physical activity	China	Adults wwith recent stroke	53 (12 vs. 62 (12)	23%	N/A	30	2	1) Feedback; 2) No feedback	ZGPAX S8 Android™ smartwatch	21 days
Lukkahatai et al., 2021 [25]	Physical activity	Thailand	Adults with diabetes	56.5 (7.2)	70.20%	Thai heritage	76	2	1) Feedback; 2) No feedback	Garmin Vivofit accelerometer	2 days
Paschali et al., 2005 [52]	Physical activity	USA	Adults with obesity & type 2 diabetes	48.8 (6.1) vs. 27.0 (7.2)	53%	N/A	30	2	1) Feedback; 2) No feedback	BioTrainer accelerometer	3 months
Prestwich et al., 2016 [48]	Physical activity	UK	Inactive adults	23.81 (11.01)	> 73.4% (Not reported: 11/124)	N/A	80	2	1) Feedback; 2) No feedback	Actigraph accelerometer, study-designed website	2 weeks
Prestwich et al., 2017 [36]	Physical activity	UK	Inactive adults	21.98 (5.97) vs. 23.09 (6.96)	74.3–77.3%	N/A	192	2	1) Feedback; 2) No feedback	Yamax CW-300 pedometer, study-designed website	5 weeks
Human- Versus Algorithm Generated Feedback											
			Included Sample					Included Conditions			
Study	Target Behavior(s)	Country	Characteristics	Age (M(SD))	% Female/ Women	Ethnicity	N	#	Description	Mode of Self-Monitoring	Duration
Beleigoli et al., 2020 [43]	Weight, diet, physical activity	Brazil	Adults with overweight/ obesity	M = 33.0–34.4	75.0–78.2%	N/A	828	2	1) Platform-only; 2) Platform + Coaching	Study-designed website	24 weeks
Kim et al., 2021 [53]	Physical activity	USA	Adults	29.7 (10.0)	41%	55.5% White	24	2	1) Tailored feedback about sedentary time; 2) Non-tailored feedback	Actigraph GT3X accelerometer, study-designed website	2 weeks
Tate et al., 2006 [41]	Weight, diet, physical activity	USA	Adults with overweight/ obesity	49.2 (9.8)	84.38%	87–90% White	125	2	1) Automated feedback; 2) Human-generated feedback	Study-designed website	6 months
West et al., 2022 [29]	Weight, diet, physical activity	USA	Adults with overweight/ obesity	50.5 (11.2)	90.40%	83.6% White	37	2	1) Pre-scripted feedback; 2) Human-generated feedback	Fitbit website/app, Renpho e-scale	16 weeks
Other Types of Feedback Comparisons											
			Included Sample					Included Conditions			
Study	Target Behavior(s)	Country	Characteristics	Age (M(SD))	% Female/ Women	Ethnicity	N	#	Description	Mode of Self-Monitoring	Duration
Ambeba et al., 2015; Burke et al., 2011; Burke et al., 2012; Conroy et al., 2011; Turk et al., 2013; Wang et al., 2012 [5, 27, 49, 50, 54, 55]	Weight, diet, physical activity	USA	Adults with overweight/ obesity	46.8 (9.0)	84.80%	78.10%	140	2	1) Weekly human feedback; 2) Weekly human feedback + Daily PDA feedback	Personal digital assistant	24 months
Godino et al., 2013 [44]	Physical activity	UK	Adults	47.5 (6.8)	53.40%	98.3% White	346	4	1) Simple feedback; 2) Visual feedback; 3) Contextualised feedback	Actiheart device	6 days
Martin et al., 2015 [26]	Physical activity, sedentary behavior	UK	Middle aged men who were sedentary	55%, 30–49 years old	0%	85% White	40	2	1) Feedback on upright time; 2) Feedback on sedentary time	SitFIT device	4 weeks
Kerrigan et al., 2021 [46]	Physical activity	USA	Adults with overweight/ obesity who were inactive	48.95 (14.20)	98.20%	89.3% White	31	2	1) Daily feedback; 2) Weekly feedback	Fitbit activity tracker	6–8 weeks
Prestwich et al., 2017 [36]	Physical activity	UK	Inactive adults	21.98 (5.97) vs. 23.94 (9.16)	22.7–24.4%	N/A	281	2	1) Basic feedback; 2) Competition Feedback	Yamax CW-300 pedometer, study-designed website	5 weeks
Rabbi et al., 2015 [47]	Physical activity, diet	USA	Adults with a mobile device	28.3 (6.96)	47%	N/A	17	2	1) Tailored feedback; 2) Generic feedback	Study-designed MyBehavior app	3 weeks

The median number of study participants was 80 (range: 17–828). The majority of studies included samples with a mean age between 30 and 50 [27, 28, 40–42, 44, 46, 51, 52] who were predominantly female [25, 27–29, 36, 40–44, 46, 48, 51, 52] and White [26–29, 41, 42, 44, 46, 51, 53] (although ethnicity or race was not reported in 7 publications). Studies were conducted in the United States (*n* = 10, 52.6%; [27–29, 41, 42, 46, 47, 51–53], Brazil (*n* = 1, 5.3% [43]), the Netherlands (*n* = 1, 5.3% [40]), Finland (*n* = 1, 5.3% [24]), Thailand (*n* = 1, 5.3% [25]), China (*n* = 1, 5.3% [45]), and the United Kingdom (*n* = 4, 21.1% [26, 36, 44, 48]. Study duration ranged from 2 days to 24 months, although most interventions (*n* = 11) were shorter than 12 weeks. The studies were published between 2005 and 2022.

### Theoretical foundation

In total, 11 studies (57.9%) cited a theoretical foundation for the intervention. The most frequently cited theories were general Behavioral Theory (*n* = 2, 10.6%), Control Theory (*n* = 2, 10.6%), and Social Cognitive Theory (*n* = 4, 21.1%).

### Retention

Retention rates were calculated by the number of participants who completed the final follow-up data collection visit (varying between studies from 2 days to 24 months). On average, retention was 76.3% (range: 48.0%-100%).

### Feedback features of included studies

The 19 studies varied in the content, frequency, and the timing of the feedback, with often limited details provided about the feedback (Table 3). Feedback was often graphical, such as a history of physical activity data, or reduced to numerical indicators for activity units or steps (e.g., [25, 48]). Some studies provided feedback on goal attainment (e.g., [43, 46]) or positive reinforcement (e.g., [29, 40]). Most studies, especially if published recently, provided feedback on digital devices such as PDAs (e.g., [27]), smartphone apps (e.g., [51]), or smartwatches [45]. Frequency of feedback varied, the majority of studies provided feedback continuously (3 studies; [25, 26, 45]), daily (or multiple times daily) (5 studies; [28, 29, 42, 47, 48]), or weekly (3 studies; [36, 41, 53]). Other studies had varied feedback frequency throughout the study (i.e., decreasing frequency) [27], randomization to different frequencies [46], or did not describe the frequency of feedback [24, 40, 43, 51].

**Table 3:** Feedback Characteristics

Study	Included Conditions Description	Self-Monitoring Focus of the Feedback	Feedback Modality	Feedback Frequency	Feedback Length	Feedback Content
Ambeba et al., 2015; Burke et al., 2011; Burke et al., 2012; Conroy et al., 2011; Turk et al., 2013; Wang et al., 2012 [5, 27, 49, 50, 54, 55]	1) Weekly human feedback; 2) Weekly human feedback + Daily PDA feedback	Progress toward goal attainment related to diet, physical activity, and self-monitoring	Conditions 1 & 2: Written human feedback; Condition 2: Additional PDA-based feedback based on an algorithm delivered at a randomly-selected time	Conditions 1 & 2: Once a week for months 1–4, bi-weekly for months 5–12, once a month for months 13–18; Condition 2: Daily	Conditions 1 & 2: Not specified; Condition 2: 1–2 sentences	Conditions 1 & 2: Not specified; Condition 2: Messages included both positive reinforcement about the participant’s progress and guidance to direct behaviors to stay within goals
Beleigoli et al., 2020 [43]	1) Platform-only; 2) Platform + Coaching	Personalized goals	Condition 1: Study-designed website; Condition 2: Private forum embedded in the study website	Not specified	Not specified	Conditions 1 & 2: Behavioral feedback and suggestions of strategies for individualized goal achievement; Condition 2: Self-monitoring promotion, emotional social support, informative social support on diet quality and quantity, guiding in the development of action plans and of problem solving strategies
Burke et al., 2017 [42]	1) Feedback; 2) No feedback	Meeting dietary calorie, fat, and sugar goals; self-monitoring adherence	Condition 1: Study-designed app; Condition 2: N/A	Condition 1: 1–4 times per day; Condition 2: N/A	Condition 1: 1–3 sentences: Condition 2: N/A	Condition 1: Percentages of calories, fat and sugar that approximated various combinations of 1) under-eating, 2) meeting goals, or 3) exceeding goals; Condition 2: N/A
Burke et al., 2022 [28]	1) Feedback; 2) No feedback	Minutes of physical activity, diet, weight	Condition 1: Study-designed app; Condition 2: N/A	Condition 1: 3 times per day; Condition 2: N/A	Condition 1: 1–2 sentences based on the example; Condition 2: N/A	Condition 1: Addressed one behavior/outcome at a time; Condition 2: N/A
Blanson Henkemans et al., 2009 [40]	1) Computer assistant feedback; 2) No computer assistant feedback	Individualized lifestyle goals	Condition 1: An animated cat will look happy or sad depending on participant goal achievement; Condition 2: N/A	Not specified	Not specified	Condition 1: Expresses empathy about other priorities, positive reinforcement, explores discrepancies between lifestyle goal and current lifestyle; encourages self-efficacy and optimism; Condition 2: N/A
Fanning et al., 2017 [51]	1) Goal-setting + Feedback points; 2) Goal setting only; 3) Feedback points only; 4) None	Conditions 1–4: Physical activity; Condition 3: In-App activities	Conditions 1–4: Study-designed app	Conditions 1–4: Graphical feedback was continuously available; Conditions 1–3: Support emails were sent twice per week	Not specified	Conditions 1–4: Graphical feedback depicting intensity, enjoyment, number of bouts, minutes of activity completed within the week, progress toward goals; Conditions 1–2: Goal setting reminders, progress toward goals, activity summary table, reminders to be active; Condition 3: Points for in-app activity, increasingly fit avatar, new achievement level titles
Godino et al., 2013 [44]	1) Simple feedback; 2) Visual feedback; 3) Contextualized feedback	Physical activity	Conditions 1-3: Mailed letter	Single occurrence	1–4 pages	Conditions 1–3: Definition of physical activity, summary of physical activity's health benefits, current physical activity guidelines, participant's current physical activity level; Condition 2: Addition of line graphs of participant's heart rate and daily movement counts; Condition 3: Addition of possible ways to increase physical activity level
Jauho et al., 2015 [24]	1) Activity tracker with feedback; 2) Activity tracker with no feedback	Physical activity	Condition 1: On the device screen; Condition 2: N/A	Condition 1: Continuous; Condition 2: N/A	N/A	Condition 1: Accumulated daily moderate-vigorous physical activity time, time spent on different physical activity levels, steps, and calories burned for each day; Condition 2: N/A
Kerrigan et al., 2021 [46]	1) Daily feedback; 2) Weekly feedback	Steps	Conditions 1 & 2: Study-designed website, text message	Conditions 1 & 2: Continuous; Condition 1: Daily; Condition 2: Weekly	1–2 sentences based on the example	Condition 1: Established at midafternoon how close the participant was to meeting the goal; Condition 2: Summary of number of days out of the previous 7 that the goal was met
Kim et al., 2021 [53]	1) Tailored feedback about sedentary time; 2) Non-tailored feedback	Sedentary time	Conditions 1 & 2: Verbal	Twice during the study period	Not specified	Conditions 1 & 2: Education, general goal setting advice; Condition 1: Tailored goal setting based on self-monitored behavior, suggestions of non-sedentary behavior
Lawrie et al., 2018 [45]	1) Feedback; 2) No feedback	Physical activity	Condition 1: On the device screen; Condition 2: N/A	Condition 1: Continuous; Condition 2: N/A	N/A	Condition 1: Visual feedback; Condition 2: N/A
Lukkahatai et al., 2021 [25]	1) Visual feedback; 2) No feedback	Steps	Condition 1: On the device screen; Condition 2: N/A	Condition 1: Continuous; Condition 2: N/A	N/A	Condition 1: Step count; Condition 2: N/A
Martin et al., 2015 [26]	1) Feedback on upright time; 2) Feedback on sedentary time	Sedentary behavior; steps	Conditions 1 & 2: On the device screen: Condition 2: Vibration related to length of sitting time	Continuous	N/A	Condition 1: Percentage of upright time in green; Condition 2: Percentage of sitting time in yellow
Paschali et al., 2005 [52]	1) Feedback; 2) No feedback	Physical activity	Condition 1: Computer screen, printed report; Condition 2: N/A	Condition 1: Once per month (3 times); Condition 2: N/A	N/A	Condition 1: Graphical display; Condition 2: N/A
Prestwich et al., 2016 [48]	1) Goal setting + Self-monitoring; 2) Goal setting + Self-monitoring + Feedback	Physical activity	Condition 1: N/A; Condition 2: Text message	Daily	N/A	Condition 1: N/A; Condition 2: Graphical feedback with labels for daily and weekly activity units
Prestwich et al., 2017 [36]	1) Feedback; 2) No feedback; 3) Competition feedback	Physical activity	Conditions 1 & 3: Study-designed website; Condition 2: N/A	Weekly	N/A	Condition 1: Graphical display; Condition 2: N/A; Condition 3: League table of their position relative to other participants
Rabbi et al., 2015 [47]	1) Tailored feedback; 2) Generic feedback	Physical activity, diet	Conditions 1 & 2: Study-designed app	Daily	4 sentences	Condition 1: Positive feedback and suggestions for dietary and physical activity changes, tailored to context and person; Condition 2: Generic diet and physical activity suggestions
Tate et al., 2006 [41]	1) Automated feedback; 2) Human-generated feedback	Self-monitoring adherence; personalized calorie goal adherence; exercise goal adherence; weight loss	Conditions 1 & 2: Email	Weekly	Not specified	Condition 1: A feedback algorithm created a computer-tailored message of support, praise, comparison of behaviors with weight loss progress; and suggested behavioral strategies to improve self-monitoring adherence, calorie and exercise goal adherence, and weight loss; Condition 2: Clinical judgement to provide feedback on weekly weight loss compared with overall progress, progress toward behavioral goals, overcoming specific weight loss barriers, motivation, and answers to participants' questions
West et al., 2021 [29]	1) Pre-scripted feedback; 2) Human-generated feedback	Dietary monitoring, physical activity, self‐weighing	Conditions 1 & 2: Email	Weekly	Condition 1: 4 short paragraphs; Condition 2: Averaged 180 words	Condition 1: Pre‐scripted messages with three options (success, partial success, absence of self‐monitoring) regarding dietary monitoring, physical activity, self‐weighing; Condition 2: Positive reinforcement for successful goal achievement related to diet, physical activity, and weight self-monitoring, identified possible areas for improvement, and suggested possible strategies for identified challenges

Across the studies, 9 compared feedback to no feedback [24, 25, 28, 36, 40, 42, 45, 48, 51, 52] and 5 compared human- versus algorithm-generated feedback [29, 41, 43, 47, 53]. The remaining 4 studies included other types of feedback comparisons, including feedback frequency (daily vs. weekly [27, 46]), richness of feedback (simple vs. visual vs. contextualized [44]), and the behavior on which feedback was provided (upright time vs. sedentary time [26]).

### Impact of feedback on diet and physical activity behaviors, weight, and self-monitoring behaviors

A file containing means and standard deviations for all group comparisons can be found on the OSF (https://osf.io/j9duf/).

### Impact of feedback provision

Nine studies compared participants who received and did not receive feedback, allowing us to test whether providing feedback had a positive impact on behaviors or weight. Studies yielded mixed results. Six studies reported benefits of feedback such as reaching diet goals [40], self-monitoring diet and exercise more frequently [40], losing more weight [40], and being more physically active [24, 36, 45, 48, 52]. This positive impact, however, was not universally observed; other comparisons did not report an impact of feedback provision on physical activity [25, 52] or weight [24, 28, 42].

**Fig. 2:**
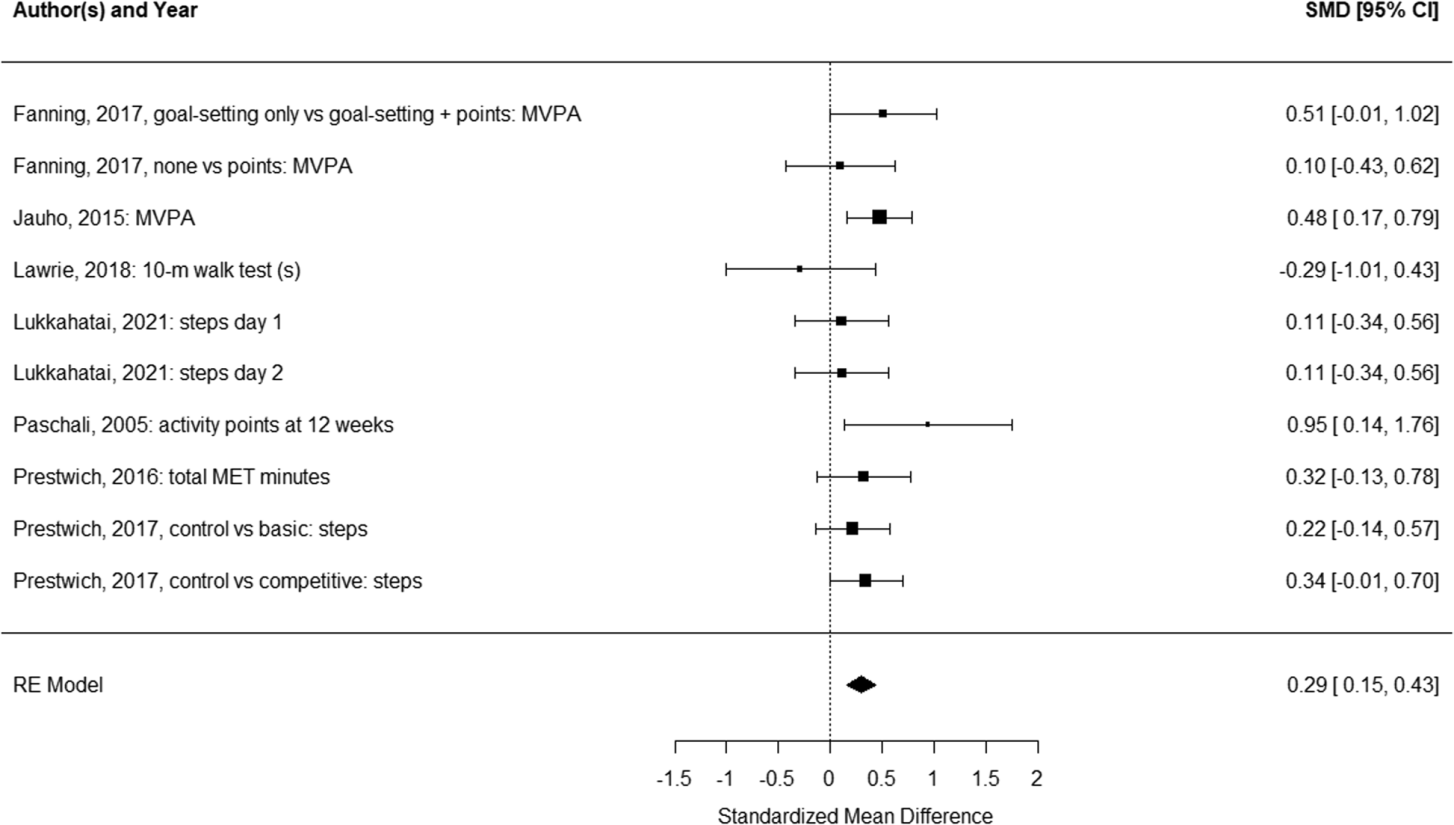
Forest plot for the random effects meta-analysis comparing the impact of providing feedback vs not providing feedback on physical activity behaviors

**Fig. 3:**
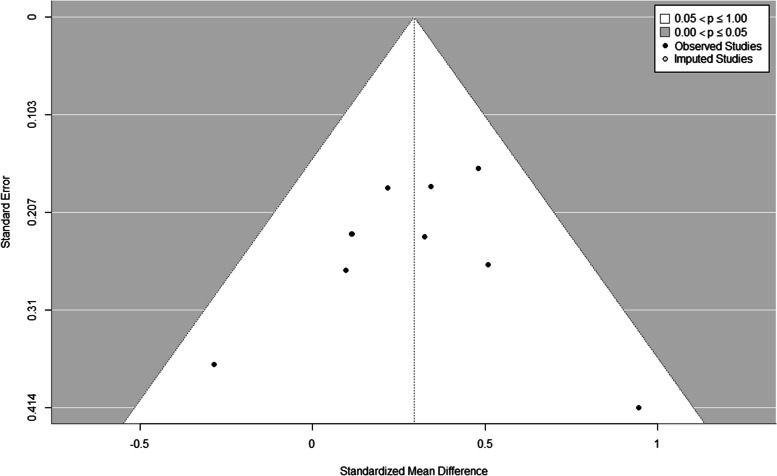
Funnel plot created using the trim-and-fill method. No studies were filled, indicating that publication bias is unlikely

Due to the large heterogeneity of studies in terms of feedback provided and outcomes studied (e.g., reporting weight change in various ways), we were only able to conduct a random effects meta-analyses for differences in physical activity based on 9 comparisons reported in 6 studies [25, 36, 45, 48, 51, 52]. The meta-analysis yielded a statistically significant pooled effect size of Cohen’s d = 0.29, 95% CI [0.16;0.43] (test for overall effect: Z = 4.14, *p* < 0.001; see Fig. 2). Heterogeneity was low (I^2^ = 9.07, Tau^2^ = 0.00, H^2^ = 1.00, *df* = 9, *p* = 0.432 [56]). Results were unchanged when using trim-and-fill, indicating no evidence for publication bias (see Fig. 3).

### Impact of human vs. algorithm-generated feedback

Five studies compared the impact of human- and algorithm-generated feedback on behaviors and weight with mixed results. Studies reported significant group differences, including healthier diet composition in participants receiving human-generated feedback [43] and a reduction in sedentary time [53]. Conversely, West et al. [29] reported greater weight loss in participants who received algorithm-generated feedback, compared to participants who received human-generated feedback. Other studies did not report differences between groups for physical activity [41, 43], self-monitoring behaviors [43], or weight loss [41]. Due to the large heterogeneity of studies in terms of feedback provided and outcomes studied, we were unable to conduct any meta-analyses.

### Impact of other forms of feedback

Five studies investigated the impact of different forms of feedback on behaviors. Due to the large heterogeneity of studies in terms of feedback provided and outcomes studied, no meta-analysis could be conducted with these studies. The impact of feedback frequency was tested in two studies, including the SMART trial that resulted in several publications (as described below). Kerrigan et al. [46] reported that providing daily feedback increased step counts more than providing weekly feedback. This finding was not mirrored by the publications stemming from the SMART trial related to weight management (i.e., the primary SMART study outcome) [27, 49] or physical activity [27]; however, the SMART trial reported a greater reduction in energy consumed for participants who received daily vs. weekly feedback messages [54] and found greater adherence to self-monitoring if daily feedback messages were provided [55]. Self-monitoring behaviors were then correlated with greater adherence to physical activity goals and weight loss [49, 50].

In a test of another type of feedback, Godino et al. [44] tested whether feedback richness (simple vs. visual vs. contextualized) impacted participants’ physical activity, and found no significant group differences. In addition, Rabbi et al. [47] tested whether personalized feedback (personalized vs. non-personalized feedback, both generated by an algorithm) affected participants’ diet and physical activity. The authors reported that personalized feedback led to increased physical activity, but dietary behaviors were not different between the conditions. Finally, Martin et al. [26] investigated if the behavior on which feedback (i.e., sedentary time vs. upright time) was provided impacted participants’ physical activity. Again, no significant group differences were found.

## Impact of feedback on participants’ perception of the intervention and retention

Seven of the included studies [25, 26, 36, 44, 47, 51, 52] reported on participants’ evaluation of the provided feedback. In all seven studies, evaluations were highly positive, with participants reporting that the feedback was motivating [26] and the main reason for using the intervention device [25, 52]. Fanning et al. [51] reported that participants asked for more frequent messages, and Paschali et al. [52] noted that participants in the “no feedback” condition were disappointed that they did not receive any feedback. Feedback thus seems to be an integral component of interventions that participants expect and enjoy. Somewhat unexpectedly, participants even reported that they found the feedback motivating and enjoyable even if it was not related with objectively measured or even perceived changes in behavior (e.g., [26, 52]).

Three of the included studies explicitly compared retention rates between conditions. All three studies [29, 41, 42] did not report differences in retention based on the condition, suggesting that feedback might not prevent attrition.

### Risk of bias assessment

We used the Cochrane Risk of Bias 2.0 tool to evaluate all of the studies. All studies were subject to significant risk of bias (see Table 4 for details), with 9 studies having the overall rating of some concern and 10 studies receiving the overall rating of high risk of bias. The high risk of bias largely resulted from lack of pre-registration of the analysis plan.

**Table 4:** Risk of bias assessment for included studies

Study	Bias arising from the randomization process	Bias due to deviations from the intended interventions	Bias due to missing outcome data	Bias in measurement of the outcome	Bias in selection of the reported result	Overall rating
Ambeba et al., 2015; Burke et al., 2011; Burke et al., 2012; Conroy et al., 2011; Turk et al., 2013; Wang et al., 2012 [5, 27, 49, 50, 54, 55]	some concerns	low risk	low risk	some concerns	high risk	high risk
Beleigoli et al., 2020 [43]	low risk	low risk	some concerns	some concerns	some concerns	some concerns
Burke et al., 2017 [42]	some concerns	low risk	some concerns	low risk	some concerns	some concerns
Burke et al., 2022 [28]	some concern	low risk	low risk	low risk	low risk	some concern
Blanson Henkemans et al., 2009 [40]	low risk	some concerns	high risk	high risk	high risk	high risk
Fanning et al., 2017 [51]	some concerns	low risk	some concerns	low risk	some concerns	some concerns
Godino et al., 2013 [44]	some concerns	some concerns	some concerns	low risk	some concerns	some concerns
Jauho et al., 2015 [24]	some concerns	low risk	some concerns	low risk	some concerns	some concerns
Kerrigan et al., 2021 [46]	some concerns	some concerns	some concerns	some concerns	high risk	high risk
Kim et al., 2021 [53]	some concerns	some concerns	some concerns	low risk	high risk	high risk
Lawrie et al., 2018 [45]	high risk	some concerns	some concerns	low risk	high risk	high risk
Lukkahatai et al., 2021 [25]	some concerns	low risk	low risk	some concerns	high risk	high risk
Martin et al., 2015 [26]	low risk	low risk	some concerns	low risk	some concerns	some concerns
Paschali et al., 2005 [52]	some concerns	low risk	some concerns	low risk	high risk	high risk
Prestwich et al., 2016 [48]	low risk	some concerns	some concerns	low risk	some concerns	some concerns
Prestwich et al., 2017 [36]	low risk	low risk	some concerns	some concerns	high risk	high risk
Rabbi et al., 2015 [47]	some concerns	low risk	low risk	low risk	high risk	high risk
Tate et al., 2006 [41]	some concerns	some concerns	low risk	some concerns	high risk	high risk
West et al., 2021 [29]	some concerns	low risk	low risk	low risk	some concerns	some concerns

## Discussion

Feedback is a core component of behavioral change interventions [17]; however, because feedback is rarely the focus of intervention and thus varied systematically, little is known about how feedback should ideally be formulated and presented. The current systematic review aimed to compile the existing evidence about feedback on self-monitoring behaviors, dietary intake, physical activity, and weight. Overall, evidence for the effectiveness of feedback was mixed. There was a significant effect for feedback (vs. no feedback) on physical activity, but this finding was driven by only half of the studies reporting a significant effect for including feedback (compared to no feedback). However, the effect of the presence or absence of feedback for outcomes other than physical activity has rarely been examined and thus we were unable to conduct meta-analyses for these other outcomes.

Despite the popularity of digital interventions which often incorporate algorithm-generated feedback [57, 58], effects of providing human- vs algorithm-generated feedback is understudied. Interestingly, while results of four out of five included studies reported either no difference or human-generated feedback to be superior, findings by West et al. [29] suggest that algorithm-generated feedback may be more effective in certain circumstances. For example, algorithms consistently provide feedback on all of the desired behaviors, which may not happen with a human, and algorithms can provide more immediate feedback, without consideration for holidays, illness, or weekends. In addition, complex algorithms may detect patterns of behavior that may be beyond the capabilities of an interventionist. More research is urgently needed to understand which form of feedback generation are most effective under which circumstances, given that generating feedback automatically may improve the cost-effectiveness and sustainability of behavioral interventions as well as their reach [20].

Available research regarding feedback frequency was especially limited. Two studies [46, 55] focused on the frequency of providing feedback, showing that daily feedback was associated with greater self-monitoring, which was in turn associated with improved behavioral and health outcomes such as physical activity and weight loss. The link between self-monitoring and intervention effectiveness has been previously established; providing feedback frequently (but also not too frequently so that it may annoy users, especially when paired with a notification [59]) may thus be key for intervention effectiveness. More research is needed to confirm these findings also for other behaviors and to determine potential dose–response effects of feedback for the engagement with intervention components.

It is important to note that there are numerous characteristics of within the design of each feedback package (e.g., frequency, behavioral vs. outcome focus, length, personalization, graphical vs. numerical vs. text vs. vibration modality, achievement vs. future behavior change valence). Due to the infrequency of each characteristic of feedback and the lack of systematic manipulation of some of these characteristics, we were not able to evaluate the independent effects of these characteristics, which may have led to the mixed outcomes in this review. It will be important to systematically vary these feedback characteristics to determine optimal combinations, as some of these characteristics may have small but potentially additive effects.

This review only included studies that specifically compared different feedback conditions and not intervention packages, to isolate effects of feedback provision and different forms of feedback. However, different BCTs included in an intervention may interact since they link to or build on one another. For example, feedback provision may boost the effectiveness of other BCTs such as goal-setting since it may allow participants to identify changes that are most urgently needed or easiest to achieve [60]. Potential interactions between BCTs may also explain why Fanning [51] (which also used goal-setting) reported relatively large effects of feedback on changes in physical activity, while other studies (which did not use goal-setting) produced smaller effects.

Based on evaluations of feedback provision reported in a small number of included studies, it can be concluded that feedback provision is a desired and well-received study component, which mirrors previous research [59]. Surprisingly, in some of these studies, feedback provision did not improve intervention effectiveness despite the study participants reporting to find it useful, perhaps because feedback sometimes focuses on what the participant is doing well and maintains a human connection in some studies. On the other hand, previous research has pointed out that feedback may not always be beneficial; depending on the valence, it may also be seen as demotivating and so promote disengagement – rather than engagement – with the intervention [61]. In addition, the studies that examined the effect of feedback on retention did not find benefits [29, 41, 42]. These findings underline that feedback needs to be carefully crafted to achieve its desired effects of promoting intervention engagement and effectiveness.

Despite the systematic approach to this review, there are limitations that are important to note. First, the details on feedback provided in studies was often unavailable, which complicates the interpretation of the findings. Second, some of the interventions were extremely short (i.e., 2 days [25]) and most interventions were less than 12 weeks, so may not have been long enough to adequately test the feedback effect. In addition, some of the outcomes we examined were too different to include in additional meta-analyses. Furthermore, many studies had to be excluded because they tested intervention packages, which makes it difficult to estimate effects of individual intervention components. Third, the vast majority of included studies did not conduct sensitivity analyses to test for potential demographic differences in effects, and many included samples that were predominantly female, well educated, and white. This review thus cannot speak to the generalizability of the findings to deprived populations. Future research needs to address this issue, since engagement with and effectiveness of behavioral interventions likely are not equal for all [62, 63]. Finally, there was a high risk of bias in the majority of the studies, reflecting changing trends in pre-registration of analyses. In the future, rigorous experimental research using appropriate study designs such as factorial trials are needed to examine optimal feedback components further.

However, there are also strengths of this study. The design and conduct of the literature searches by an experienced medical librarian, the inclusion of 5 literature databases, and the use of forward and backward citation searches, which led to a comprehensive set of literature upon which to perform the review. Additionally, consistent with open science principles, we have reported the raw data on the OSF website. Finally, two reviewers independently coded all of the studies.

## Conclusion

This review underlines the importance of feedback as a behavior change technique in interventions, but also clearly indicates that greater detail should be provided in scientific manuscripts regarding the feedback components (including examples and potentially screenshots) and frequency. In addition, more research is needed on how feedback is best generated (i.e., what can be generated by an algorithm and what potentially cannot) and presented to maximize intervention effectiveness.

### Supplementary Information

**Additional file 1. **

## Data Availability

Raw data and analysis scripts are provided on the Open Science Framework (OSF; https://osf.io/j9duf/).
