# Correction: Impact of feedback generation and presentation on self-monitoring behaviors, dietary intake, physical activity, and weight: a systematic review and meta-analysis

**DOI:** 10.1186/s12966-024-01569-8

**Published:** 2024-02-23

**Authors:** Rebecca A. Krukowski, Andrea H. Denton, Laura M. König

**Affiliations:** 1 Department of Public Health Sciences, University of Virginia, PO Box 800765, Charlottesville, VA 22908-0765 USA; 2 University of Virginia, Claude Moore Health Sciences Library, Charlottesville, VA USA; 3 Faculty of Life Sciences: Food, Nutrition and Health, University of Bayreuth, Kulmbach, Germany; 4 Faculty of Psychology, University of Vienna, Vienna, Austria

**Correction: Int J Behav Nutr Phys Act 21, 3 (2024)**

**https://doi.org/10.1186/s12966-023-01555-6**

Following the publication of the original article [1], the authors reported they made an error in using two standard errors instead of standard deviations in their meta-analysis calculations. The authors updated the meta-analysis and thus updated the text and figures accordingly. The errors and corrections are as follows:

**Table:** 

Section	Errors	Corrections
Abstract	A random effects meta-analysis indicated that physical activity interventions with feedback provision were more effective than physical activity interventions without feedback (d = 0.73, 95% CI [0.09;1.37])	A random effects meta-analysis indicated that physical activity interventions with feedback provision were more effective than physical activity interventions without feedback (d = 0.29, 95% CI [0.16;0.43])
Data extraction and synthesis	In addition, a meta-analysis was conducted if at least three studies using similar manipulations and reporting on the same outcome provided data on group means and standard deviations that could be used to calculate Cohen’s d [31]	In addition, a meta-analysis was conducted if at least three studies using similar manipulations and reporting on the same outcome provided data on group means and standard deviations or standard errors that could be used to calculate Cohen’s d [31]
Impact of feedback provision	The meta-analysis yielded a statistically significant pooled effect size of Cohen’s d = 0.73, 95% CI [0.09; 1.37] (test for overall effect: Z = 2.23, *p* = 0.026; see Fig. 2). Heterogeneity was considerable (I2 = 93.22%, Tau2 = 0.88, H2 = 14.74, *df* = 8, *p* < 0.001 [56];)	The meta-analysis yielded a statistically significant pooled effect size of Cohen’s d = 0.29, 95% CI [0.16;0.43] (test for overall effect: Z = 4.14, *p* < 0.001; see Fig. 2). Heterogeneity was low (I^2^ = 9.07, Tau^2^ = 0.00, H^2^ = 1.00, *df* = 9, *p* = 0.432 [56])
Discussion	There was a significant effect for feedback (vs. no feedback) on physical activity, but this finding was driven by only half of the studies reporting a significant effect for including feedback (compared to no feedback), out of which two [48, 51] reported very large effects compared to very small to small effects of the other studies	There was a significant effect for feedback (vs. no feedback) on physical activity, but this finding was driven by only half of the studies reporting a significant effect for including feedback (compared to no feedback)
	Potential interactions between BCTs may also explain why Fanning et al. and Prestwich et al. [48, 51] (both of which also used goal-setting) reported relatively large effects of feedback on changes in physical activity, while other studies (which did not use goal-setting) produced smaller effects	Potential interactions between BCTs may also explain why Fanning [51] (which also used goal-setting) reported relatively large effects of feedback on changes in physical activity, while other studies (which did not use goal-setting) produced smaller effects

There are also errors in Figures as follows:

Figure 2
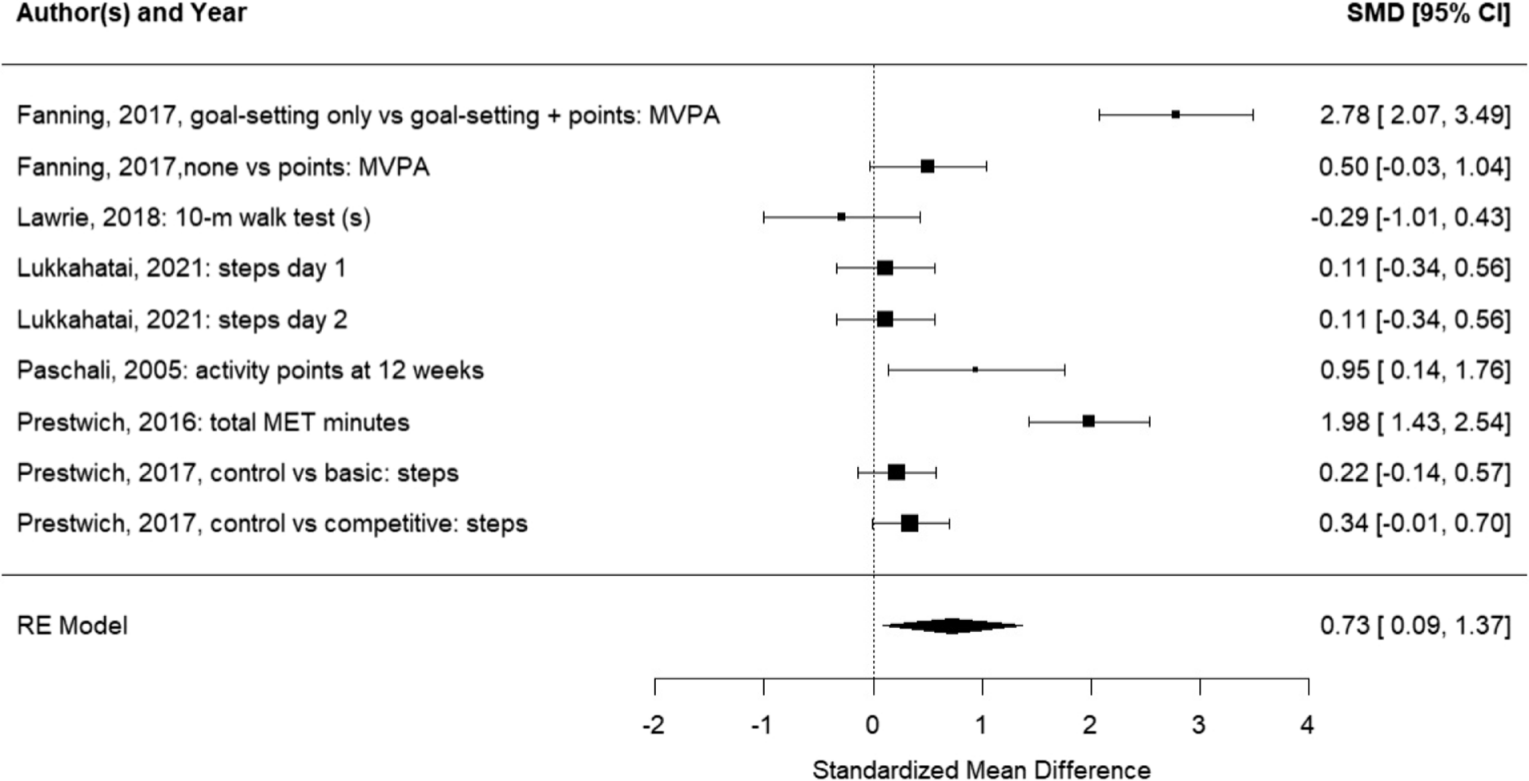


Figure 3 
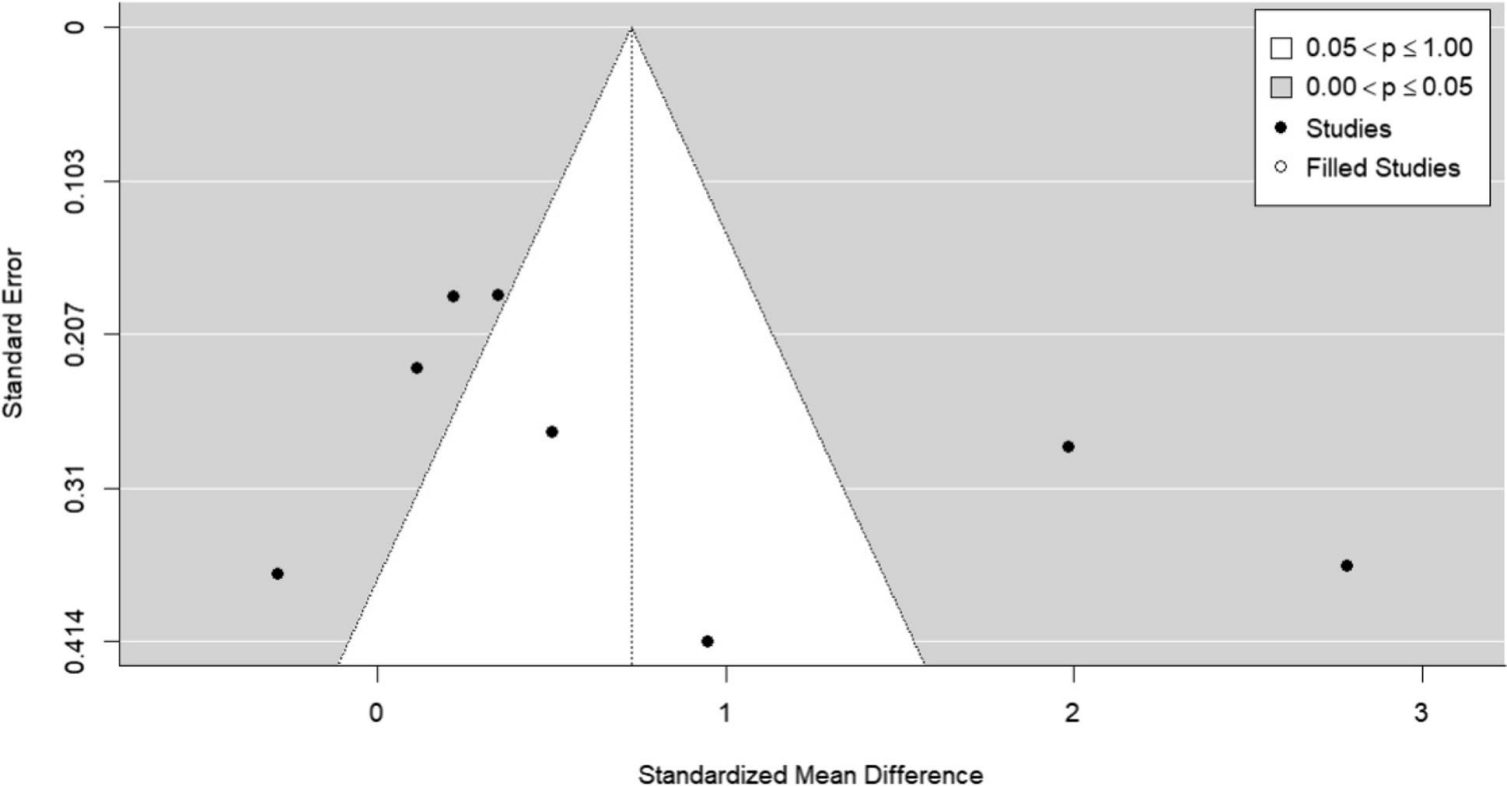


The correct figures are as follows:

**Fig. 2:**
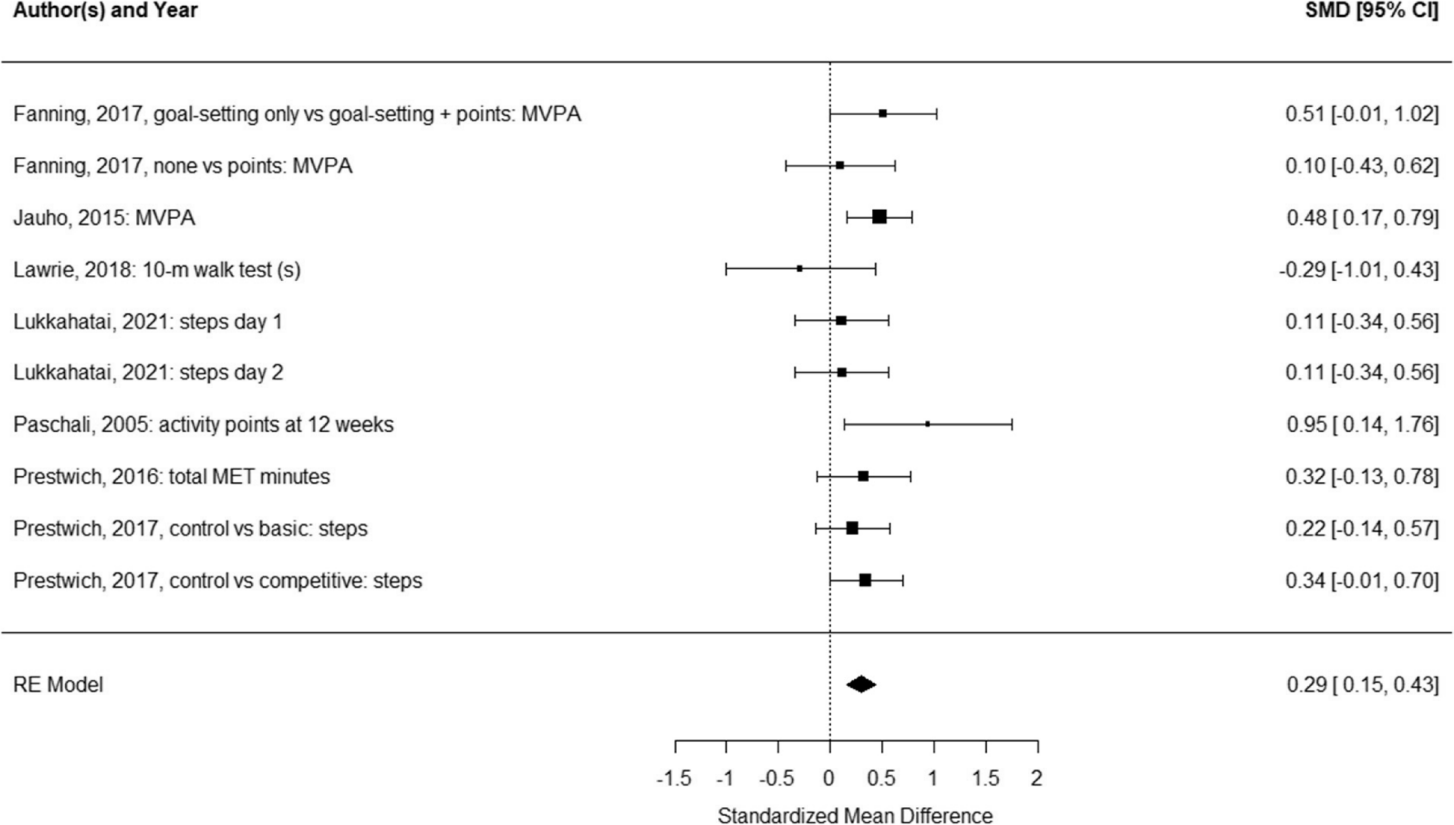
Forest plot for the random efects meta-analysis comparing the impact of providing feedback vs not providing feedback on physical activity behaviors

**Fig. 3:**
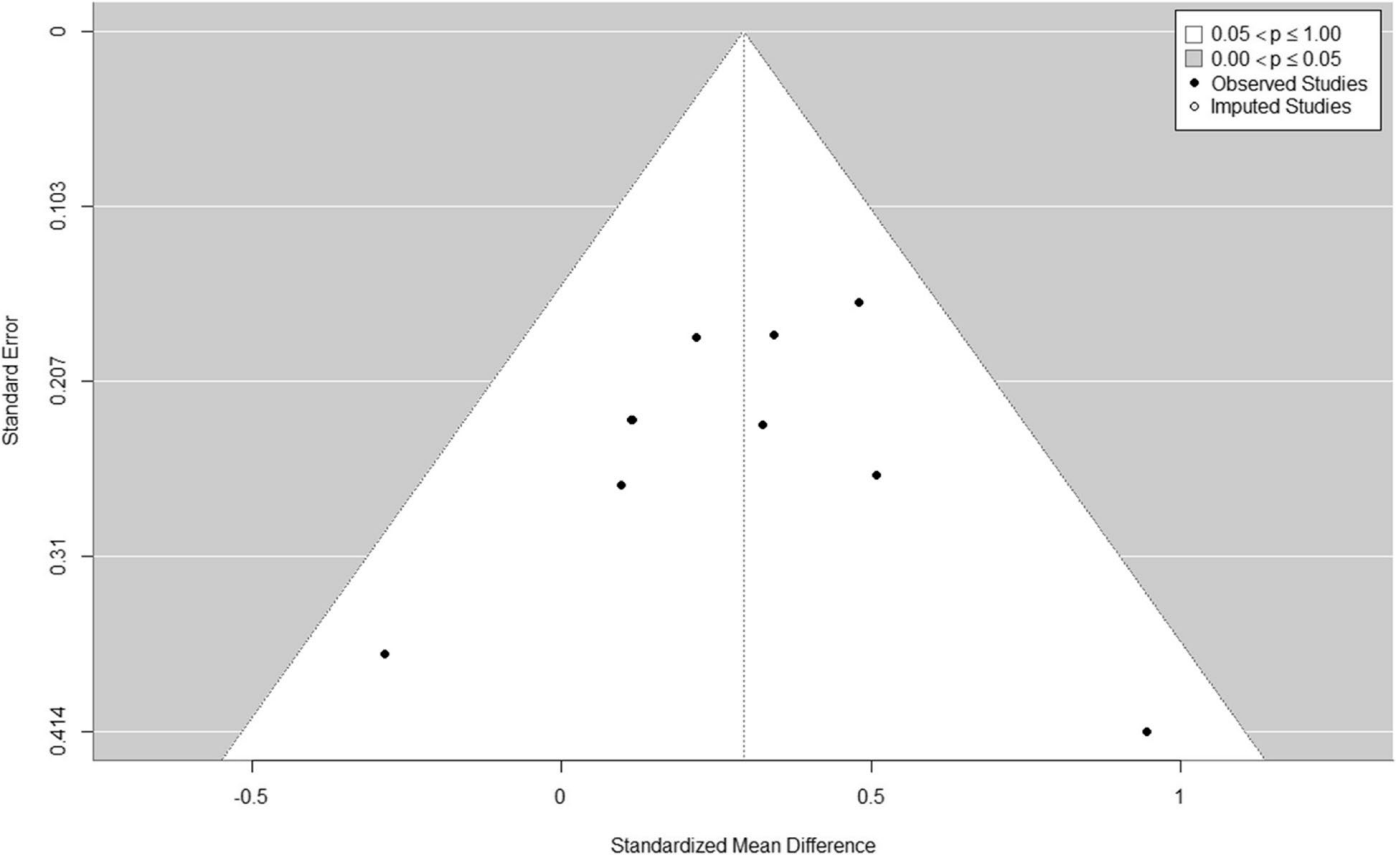
Funnel plot created using the trim-and-fll method. No studies were flled, indicating that publication bias is unlikely

The original article [1] has been updated.
